# Transcriptional Immune Signatures of Alveolar Macrophages and the Impact of the NLRP3 Inflammasome on Porcine Reproductive and Respiratory Syndrome Virus (PRRSV) Replication

**DOI:** 10.3390/v12111299

**Published:** 2020-11-12

**Authors:** Julie A. Hicks, Dongwan Yoo, Hsiao-Ching Liu

**Affiliations:** 1 Department of Animal Science, North Carolina State University, Raleigh, NC 27607, USA; jahicks3@ncsu.edu; 2 Department of Pathobiology, University of Illinois at Urbana-Champaign, Urbana, IL 61801, USA; dyoo@illinois.edu

**Keywords:** PRRSV, RNA-seq, macrophage, NLRP3 inflammasome

## Abstract

Porcine Reproductive and Respiratory Syndrome (PRRS) is a contagious viral (PRRSV) disease in pigs characterized by poor reproductive health, increased mortality, and reductions in growth rates. PRRSV is known to implement immuno-antagonistic mechanisms to evade detection and mute host responses to infection. To better understand the cellular immunosignature of PRRSV we have undertaken transcriptome and immunomodulatory studies in PRRSV-infected porcine alveolar macrophages (PAMs). We first used genome-wide transcriptome profiling (RNA-seq) to elucidate PRRSV-induced changes in the PAM transcriptome in response to infection. We found a number of cellular networks were altered by PRRSV infection, including many associated with innate immunity, such as, the NLRP3 inflammasome. To further explore the role(s) of innate immune networks in PRRSV-infected PAMs, we used an NLRP3-specific inhibitor, MCC950, to identify the potential functionality of the inflammasome during PRRSV replication. We found that PRRSV does quickly induce expression of inflammasome-associated genes in PAMs. Treatment of PAMs with MCC950 suggests NLRP3 inflammasome activation negatively impacts viral replication. Treatment of PAMs with cell culture supernatants from macrophages subjected to NLRP3 inflammasome activation (via polyinosinic-polycytidylic acid (poly I:C) transfection), prior to PRRSV infection resulted in significantly reduced viral RNA levels compared to PAMs treated with cell culture supernatants from macrophages subjected to NLRP3 inflammasome inhibition (MCC950 treatment/poly I:C transfection). This further supports a role for NLRP3 inflammasome activation in the innate macrophagic anti-PRRSV immune response and suggests that PRRSV is sensitive to the effects of NLRP3 inflammasome activity. Taken together, these transcriptome and immunoregulatory data highlight the complex changes PRRSV infection induces in the molecular immune networks of its cellular host.

## 1. Introduction

Porcine Reproductive and Respiratory Syndrome Virus (PRRSV) is a highly contagious ss (+) RNA virus, which is not currently well controlled by vaccines and other preventative strategies [1]. As such, PRRS-associated economic losses, including reproductive failure, piglet loss, and treatment measures, cost the swine industry millions of dollars annually [2]. A major roadblock to current PRRS vaccination and disease prevention strategies is PRRSV’s ability to both evade and directly antagonize the host’s immune response upon infection. Upon PRRSV infection, only a weak immunological response is mounted during the first days and weeks post-infection, which ultimately results in both a persistent viral infection and increased susceptibility to secondary infections [3]. Currently available vaccines often display the same delayed immunogenicity as field isolates and typically only provide protection against genetically-related viral strains [3]. There are several commercially available PRRSV vaccines. Among them, is the modified live virus vaccine Ingelvac MLV produced by Boehringer Ingelheim Vetmedica, Inc., St. Joseph, MO, USA. This vaccine was derived from the serial passage of one of the first PRRSV strains isolated in North America, VR-2332 (Reviewed by [4]). Molecular and genetic analyses have revealed that the Ingelvac MLV strain and VR-2332 are remarkably similar in sequence with less than 50 nucleotides differing between them [4]. Additionally, a few amino acid differences have been found within the structural genes [4].

Initial immune responses to viral infections are typically triggered by pattern recognition receptors (PRRs), such as TLRs and RLRs which detect a variety of pathogen-derived ligands. Engagement of PRRs triggers a number of immunomodulatory cascades. Among these is activation of the innate immune responder the NLRP3 inflammasome. The NLRP3 inflammasome is a multimeric complex predominantly found in macrophages, but can also be expressed by other immune cells, including dendritic cells, B-cells and T-cells (Reviewed by [5]). Inflammasomes consist of three major components: (1) either a Nod-like receptor (NLR; NLRP1, NLRP3, NLRC4) or absent in melanoma 2 (AIM2), (2) an adaptor protein apoptosis-associated speck-like protein (ASC), and (3) caspase-1 [5]. The NLRP3-mediated inflammasome is the most studied inflammasome and has been linked not only to the initial response to pathogenic infections, but also to a number of chronic inflammatory pathologies (Reviewed by [6]). Engagement of PRRs triggers NF-κB activation which in turn, induces NLRP3 expression as well as the proproteins for IL1β and IL18. NLRP3 (the sensor component) then detects a danger signal, including foreign nucleic acids, toxins, or excess ATP, which leads to the recruitment of ASC and pro-caspase-1 [6]. The newly formed inflammasome first proteolytically processes pro-caspase-1, and the now active caspase-1 then processes pro-IL1β and pro-IL18, into their active forms [6]. Mature IL1β and IL18 are then secreted to trigger a cascade of inflammatory responses. Activation of caspase-1 can also trigger pyroptosis, a caspase-1-dependent specific form of apoptosis [6].

As the NLRP3 inflammasome is an integral component of the initial antiviral response, it is not surprising that viruses have developed countermeasures to circumvent its activation. Influenza A can antagonize NLRP3 inflammasome activation by blocking post-translational modification of ASC via the virally-encoded NS1 protein, which prevents proper inflammasome assembly [7]. Sendai virus inhibits NLRP3 inflammasome activation by blocking its assembly via a direct interaction between the virally-encoded V protein and the NLRP3 protein [8]. The V protein of Measles virus also interacts with the NLRP3 protein to prevent inflammasome activation [9]. Interaction between the NLRP3 protein and the C protein of human parainfluenza virus type 3 also prevents inflammasome assembly and its subsequent activation [10]. Other viruses have evolved mechanisms to utilize NLRP3 inflammasome activation to their advantage, including HIV, infection of which is enhanced by inflammasome activation due to increased production of permissive cells [11].

PRRSV-induced alterations in the immune effector landscape of infected cells have far-reaching and long-term consequences on the host’s immune health, which affect both its ability to clear the virus and its susceptibility to secondary infections [12]. The expression of immune effectors must be tightly regulated in order to maintain homeostatic immune activities and also quickly and appropriately respond to immune triggers. Disruption of these regulatory processes by invading pathogens, such as PRRSV, can be detrimental to host immunity. To thoroughly characterize macrophagic pathways associated with PRRSV infections we employed RNA-seq to develop global transcriptome profiles of PRRSV-infected PAMs. Notably, we found that a number of genes associated with the NLRP3 inflammasome response were altered in PRRSV infected PAMs. As the NLRP3 inflammasome is a well-known branch of innate immunity, particularly in macrophages, we then used a NLRP3 inflammasome inhibitor, MCC950, which specifically and potently blocks processing of pro-caspase-1 proprotein into its mature form [13], to assess the potential involvement of the NLRP3 inflammasome in cell response to and replication of PRRSV. We found that PRRSV is likely sensitive to the effects of NLRP3 inflammasome activation.

## 2. Materials and Methods

### 2.1. Cells and Virus

Primary alveolar macrophages (PAMs) were collected via bronchoalveolar lavage from three 7-week old pigs. All animal protocols were approved by the North Carolina State University Institutional Animal Care and Use Committee (IACUC#12-079-B) and were performed in accordance with the United States Department of Agriculture Animal Welfare Act guidelines. PAMs were cultured in RPMI 1640 (Corning Life Sciences, Tewksbury, MA, USA) supplemented with L-glutamine (Sigma-Aldrich, St. Louis, MO, USA), penicillin (100 U/mL) (Sigma-Aldrich), streptomycin (100 μg/mL) (Sigma-Aldrich) and fungizone (4 μg/mL) (Sigma-Aldrich) and 10% fetal bovine serum (FBS) (Atlanta Biologicals, Flowery Branch, GA, USA). PRRSV strain VR-2332 was obtained from ATCC (Manassas, VA, USA). The Ingelvac PRRS MLV modified life virus vaccine (Boehringer Ingelheim, Ingelheim am Rhein, Germany) was obtained from a commercial supplier (JRG Supply, Fort Dodge, IA, USA). Virus stocks were produced in MARC-145 cells. Virus titers were determined in PAMs using the Reed-Muench method and immunoflouresence staining of the viral nucleocapsid protein (SDOW-17, Rural Technologies, INC., Brookings, SD, USA).

### 2.2. RNA-Seq

A total of 2 × 10^7^ PAMs (*n* = 3 pigs) per plate (60 mm) were maintained for 24 h at 37 °C with 5% CO_2_ and then either mock infected or infected with VR-2332 at a multiplicity of infection of 1 (M.O.I. 1) in duplicate/time-point + treatment and maintained at 4 °C for 4 h. Cells were washed with 1× PBS and fresh media was added and cells were maintained at 37 °C with 5% CO_2_. Cells were collected in Tri-Reagent (Sigma-Aldrich) at 0 h post infection (hpi), 12, 24, or 48 hpi. Duplicate plates were pooled, and total RNA was purified following the manufacturer’s instructions protocol. RNA quantity and quality were assessed using an Agilent Technologies 2100 Bioanalyzer (Santa Clara, CA, USA) with a high sensitivity RNA chip. All RNA samples were high quality with a RNA integrity number (RIN) values >9. RNA-seq libraries were generated using a TruSeq RNA library preparation kit v2 (Illumina, San Diego, CA, USA) and barcode indices following the manufacturer’s instructions. The quality and quantity of the libraries were assessed on an Agilent Technologies 2100 Bioanalyzer using a high sensitivity DNA chip. Equal molar amounts of the libraries were pooled and 50bp single-end sequenced at DHMRI (Kannapolis, NC, USA) using an Illumina HiSeq 2500 sequencer.

All FASTQ sequencing files have been deposited to the National Institutes of Health Short Read Archive (NIH-SRA) (accession numbers: SAMN11874909-SAMN11874929; https://www.ncbi.nlm.nih.gov/sra/PRJNA545125). All sequencing data processing and analyses were performed using CLC genomics workbench software (Qiagen, Germantown, MD, USA) [14]. Briefly, FASTQ files were imported into the CLC genomics workbench software. The next generation sequencing (NGS) trim tool was used to remove any residual adaptor sequences and/or low quality sequences (Phred < 20). Reads were then mapped to the *Sus scrofa* reference genome (Sscrofa11.1) and normalized using the transformation and normalization tool. Differential expression was performed using the RNA-seq analysis suite, which is based on the multi-factorial EdgeR method, which assumes that the read counts follow a Negative Binomial distribution [15]. To confirm the RNA-seq results a select group of differentially expressed genes were validated using RT-qPCR (Figure S1). Pairwise comparisons were made between mock-infected and VR-2332 infected PAMs at each time point. Ingenuity Pathway Analysis (Qiagen) was then performed on the differential expression data using the integrated plugin to identify differentially expressed pathways and biological pathways and to generate network figures.

### 2.3. NLRP3 Inflammasome Inhibition

#### 2.3.1. Pretreatment of PAMs with the NLRP3 Inhibitor MCC950

A total of 2 × 10^6^ PAMs (*n* = 3 pigs) were seeded per well/12-well plate and were maintained for 24 h at 37 °C with 5% CO_2_. All treatments were carried out in duplicate. For MCC950 treatment: PAMs were treated with MCC950 (dissolved in DMSO; EMD Millipore, Burlington, MA, USA) at a final concentration of 10 μM for one hour at 37 °C prior to infection/transfection. Mock-treated groups were treated with an equal volume (50 µL/100 mL media) of DMSO. Following MCC950 treatment, PAMs were either infected with VR-2332 (M.O.I. 1), Ingelvac MLV (M.O.I. 1), transfected with 1 μg of poly I:C (polyinosinic-polycytidylic acid) (EMD Millipore) using Fugene6 (Promega, Madison, WI, USA) following the manufacturer’s protocol or mock infected/transfected. Poly I:C served as a positive control, as it is a well-known activator of the NLRP3 inflammasome [16]. At 3, 6, and 9 h post-infection (hpi)/transfection (hpt), PAMs were collected in Tri-Reagent (Sigma-Aldrich, St. Louis, MO, USA) and total RNA was purified and subjected to on-column DNase treatment using a RNA Clean and Concentrator kit (Zymo Research, Irvine, CA, USA) following the manufacturer’s protocol. RNA quality was assessed using agarose electrophoresis. One microgram of DNase-treated total RNA per sample was reverse transcribed using a SuperScript III kit (Thermo Fisher Scientific, Waltham, MA, USA) following the manufacturer’s instructions. Primers used for RT-qPCR are provided in Table S1. Primers were designed using primer-BLAST (https://www.ncbi.nlm.nih.gov/tools/primer-blast/), and when possible, such that one of the primers spanned an exon–exon junction. Each RT-qPCR reaction contained 10 ng cDNA, 1X iQ SYBR Green Supermix (Bio-Rad, Hercules, CA, USA) and 500 nM of each forward and reverse primer. RT-qPCR conditions were as follows: 95 °C for 5 min, followed by 40 cycles of 95 °C for 10 s, then 58 °C for 20 s. All reactions were performed in duplicate. Melting curve analysis was utilized to confirm gene-specific amplification. Threshold cycle (Ct) values were normalized to the expression levels of Ribosomal protein L4 (*RPL4*). Relative expressions were determined using the 2^−∆∆Ct^ method [17]. Secreted IL1B levels in cell culture supernatants were quantified using an enzyme-linked immunosorbent assay kit (ELISA) for porcine IL1B (MyBioSource, San Diego, CA, USA), following the manufacturer’s instructions. Significant differences (*p* < 0.05) in expression were determined using analysis of variance SAS software (SAS Institute, Cary, NC, USA).

#### 2.3.2. Impact of Disrupted NLRP3 Inflammasome-Induced IL1B Secretion on PRRSV Replication

In a second experiment 5 × 10^6^ PAMs (*n* = 3 pigs) were seeded per well/6-well plate and were maintained for 24 h at 37 °C with 5% CO_2_. PAMs were either treated with MCC950, at a final concentration of 10 µM, or mock-treated for 1 h at 37 °C. PAMs were then transfected with 1 μg of poly I:C (EMD Millipore) using Fugene6 (Promega, Madison, WI, USA) following the manufacturer’s protocol or mock transfected, resulting in four treatment groups: MCC950 and poly I:C treated, MCC950 only, poly I:C only, and mock-treated/transfected (negative control). At 24 hpt cell culture supernatants were collected. Pig-matched PAMs (2 × 10^6^ cells per/well/12-well plate) were then treated with 400 µL of cell culture supernatants from one of the four groups for 1 h at 37 °C and then infected with either VR-2332 (M.O.I. 1) or Ingelvac MLV (M.O.I. 1). At 6, 12, 24, and 48 h post-infection, PAMs were collected in Tri-Reagent (Sigma-Aldrich, St. Louis, MO, USA) and total RNA was purified and subjected to on-column DNase treatment using a RNA Clean and Concentrator kit (Zymo Research, Irvine, CA, USA) following the manufacturer’s protocol. One microgram of DNase-treated total RNA per sample was reverse transcribed using a SuperScript III kit (Thermo Fisher Scientific, Waltham, MA, USA) following the manufacturer’s instructions. PRRSV RNA levels were quantified using RT-qPCR as described above. Secreted IL1B levels in cell culture supernatants were quantified using an enzyme-linked immunosorbent assay kit (ELISA) for porcine IL1B (MyBioSource), following the manufacturer’s instructions. Significant differences (*p* < 0.05) in expression were determined using analysis of variance (SAS).

## 3. Results

### 3.1. Transcriptome Profiling of PRRSV Strain VR-2332 Infected Porcine Alveolar Macrophages

To develop an unbiased global view of PRRSV-induced alterations in the PAM transcriptome we utilized RNA-seq to compare gene expression changes in PAMs at 12, 24, and 48 hpi following infection with PRRSV strain VR-2332 compared to mock-infected cells. The number of trimmed mappable reads per library ranged from 6,108,744 to 10,149,147. At 12 hpi the expression of 110 genes was significantly affected (*p* < 0.05) in PRRSV-infected PAMs compared to time matched mock-infected cells. Of these affected genes, 64 were upregulated and 46 were downregulated. At 24 hpi the expression of 354 genes was significantly different between PRRSV-infected and mock-infected PAMs. Of this set of affected genes, 165 were upregulated and 189 were downregulated. At 48 hpi the number of differentially expressed genes increased to 532, with 327 having increased expression and 205 having decreased expression. Ingenuity Pathway Analysis (IPA) of these differentially expressed genes found that multiple canonical immune pathways are altered in PRRSV-infected macrophages. These pathways include: LPS/IL-1 mediated inhibition of RXR function; TNFR2 signaling; Role of Pattern Recognition Receptors in Recognition of bacteria and viruses; IL-10 signaling; NF-κB Activation by Viruses; and Toll-like receptor signaling (Table 1).

**Figure 1:**
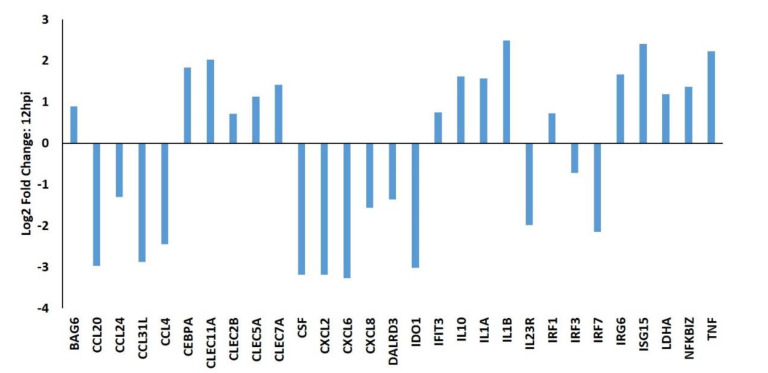
A selection of differentially expressed genes in PRRSV-infected macrophages at 12 h post infection (hpi). PAMs (*n* = 3) were either infected with PRRSV strain VR-2332 or mock infected and total RNA was collected at 12 h and subjected to RNA-sequencing. All values are provided as the log_2_ fold differences of gene expression levels in PRRSV-infected PAMs relative to mock-infected PAMs. All genes shown were statistically significantly different false discovery rate (FDR)-corrected *p* < 0.05 between the groups.

At 12 hpi many immune-related genes expressed in PAMs were already significantly (*p* < 0.05) altered by PRRSV infection (Figure 1). Among these are multiple c-type lectins including *CLEC11A* (log_2_ 2.03), *CLEC2B* (log_2_ 0.72), *CLEC5A* (log_2_ 1.12), and *CLEC7A* (log_2_ 1.41), which were all upregulated in PRRSV-infected macrophages compared to mock-infected cells (Figure 1). The transcript levels of several cytokines were upregulated in PRRSV-infected macrophages including, *IL1A* (log_2_ 1.57), *ILB* (log_2_ 2.49), and *IL10* (log_2_ 1.61), while many others were significantly downregulated in PRRSV-infected macrophages, *CCL20* (log_2_ −2.98), *CCL24* (log_2_ −1.31), *CCL31L* (log_2_ −2.87), *CCL4* (log_2_ −2.45), *CXCL6* (log_2_ −3.28), and *CXCL8* (log_2_ −1.57) (Figure 1).

**Figure 2:**
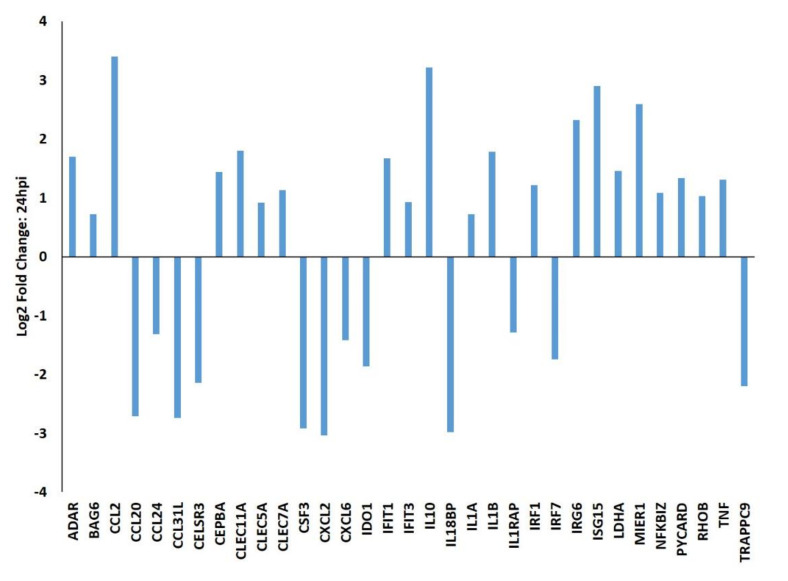
A selection of differentially expressed genes in PRRSV-infected macrophages at 24 hpi. PAMs (*n* = 3) were either infected with PRRSV strain VR-2332 or mock infected and total RNA was collected at 24 h and subjected to RNA-sequencing. All values are provided as the log_2_ fold differences of gene expression levels in PRRSV-infected PAMs relative to mock-infected PAMs. All genes shown were statistically significantly different (FDR-corrected *p* < 0.05) between the groups.

At 24 hpi, PRRSV-infected PAMs had altered expression of multiple genes involved in immune signaling, mitochondrial metabolic processes, and/or endoplasmic reticulum-trafficking (Figure 2). Among these were the upregulated c-type lectins *CLEC11A* (log_2_ 1.80), *CLEC5A* (log_2_ 0.92), and *CLEC7A* (log_2_ 1.13). Metabolism-associated genes affected by PRRSV included *IDO1* (log_2_ −1.87) and *LDHA* (log_2_ 1.46). Genes whose proteins are involved in ER-trafficking include *BAG6* (log_2_ 0.72) and *RHOB* (log_2_ 1.03), which were both upregulated in PRRSV-infected macrophages.

**Figure 3:**
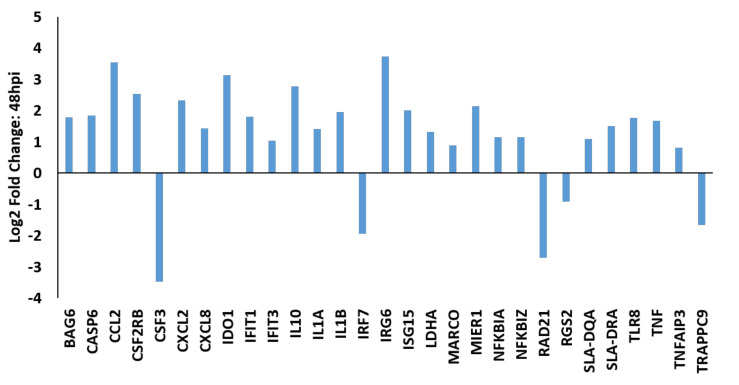
A selection of differentially expressed genes in PRRSV-infected macrophages at 48 hpi. PAMs (*n* = 3) were either infected with PRRSV strain VR-2332 or mock infected and total RNA was collected at 48 h and subjected to RNA-sequencing. All values are provided as the log_2_ fold differences of gene expression levels in PRRSV-infected PAMs relative to mock-infected PAMs. All genes shown were statistically significantly different (FDR-corrected *p* < 0.05) between the groups.

**Figure 4:**
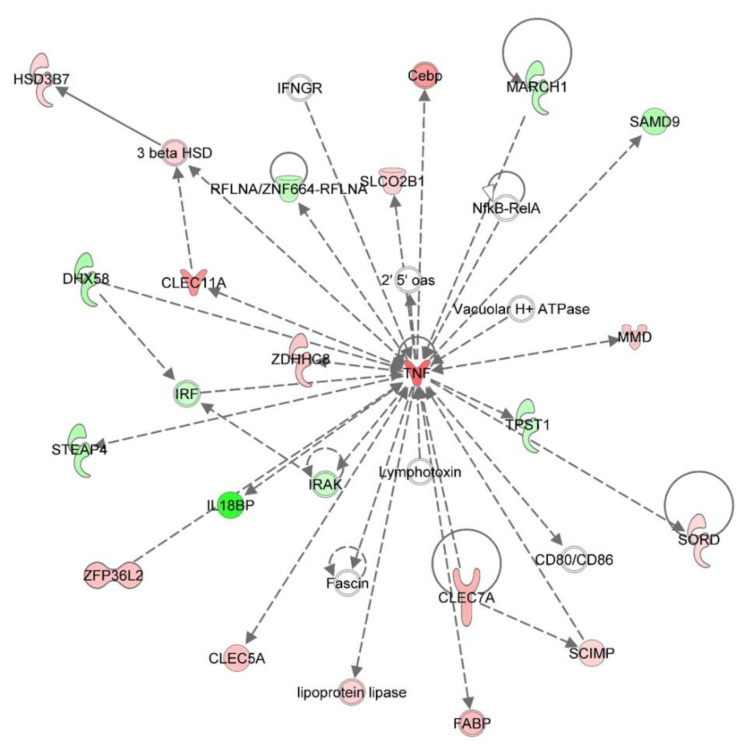
PRRSV-induced changes in the c-type lectin receptor signaling system of alveolar macrophages. Red molecules are significantly upregulated in PRRSV-infected macrophages relative to mock-infected cells and green molecules are downregulated in at least one time point (12, 24, and 48 hpi). White molecules were not significantly (*p* > 0.05) different between the two groups.

**Figure 5:**
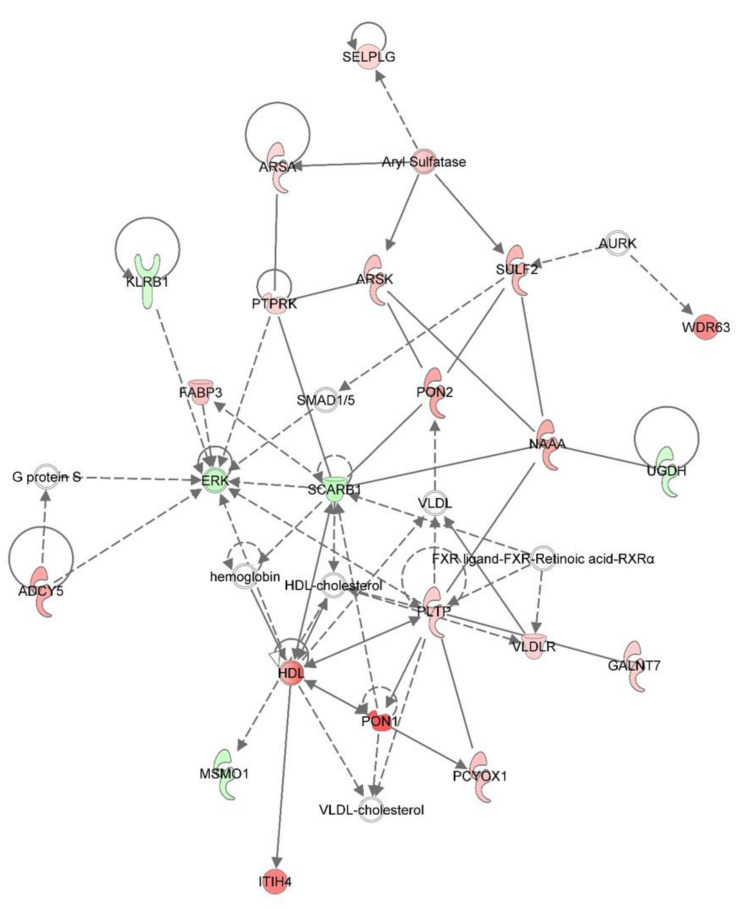
Changes in metabolic processes in PRRSV-infected alveolar macrophages. Red molecules are significantly upregulated in PRRSV-infected macrophages relative to mock-infected cells and green molecules are downregulated in at least one time point (12, 24, and 48 hpi). White molecules were not significantly (*p* > 0.05) different between the two groups.

**Figure 6:**
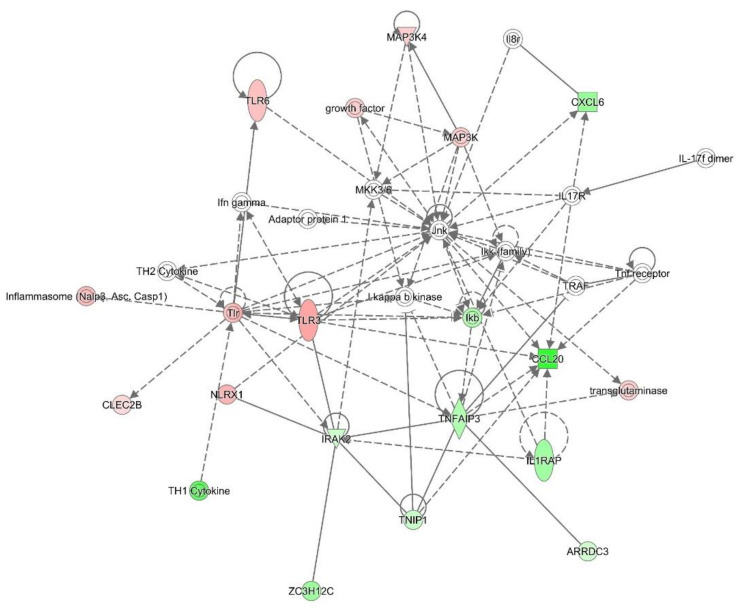
Impact of PRRSV infection on inflammasome and TLR signaling in alveolar macrophages. Red molecules are significantly upregulated in PRRSV-infected macrophages relative to mock-infected cells and green molecules are downregulated in at least one time point (12, 24, and 48 hpi). White molecules were not significantly (*p* > 0.05) different between the two groups.

PRRSV-infected macrophages had the largest alterations in their transcriptome at 48 hpi. Over 500 hundred genes were differentially expressed between PRRSV-infected PAMs and those that were mock-infected. Among these genes were the immunomodulators, *CSF3* (log_2_ −3.47), *CSF2RB* (log_2_ 2.53), *MARCO* (log_2_ 0.88), *TLR8* (log_2_ 1.76), and *CASP6* (log_2_ 1.83) (Figure 3). Two major histocompatibility complex (MHC) class II molecules, *SLA-DQA* (log_2_ 1.09) and *SLA-DRA* (log_2_ 1.50) were significantly higher at 48 hpi in PRRSV-infected macrophages relative to mock-infected cells (Figure 3). Ingenuity pathway analysis of differentially expressed genes identified in RNA-seq analysis of the PRRSV-infected PAM transcriptome revealed many immune signaling and metabolic processes are impacted by PRRSV (Figure 4, Figure 5 and Figure 6).

### 3.2. Impact of NLRP3 Inflammasome Inhibition on the PRRSV

**Figure 7:**
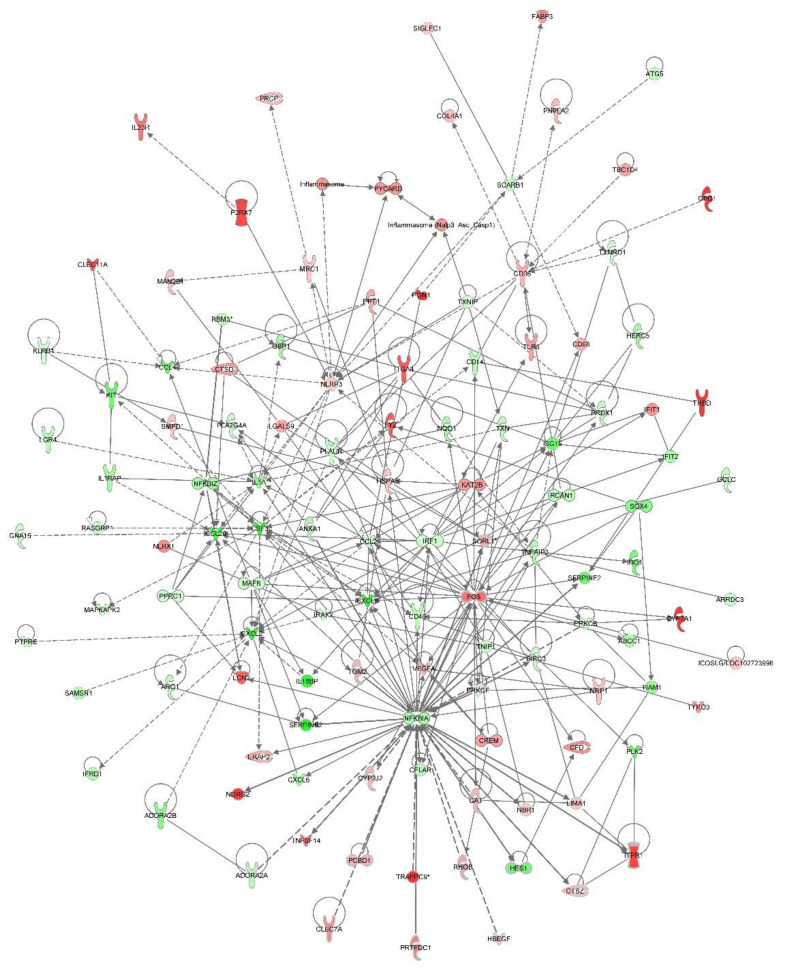
NLRP3 Inflammasome-associated genes differentially expressed in PRRSV-infected PAMs. All genes shown were significantly (*p* < 0.05) in PRRSV-infected PAMs in at least one time point (12, 24, and 48 hpi). Red molecules are significantly upregulated in PRRSV-infected macrophages relative to mock-infected cells and green molecules are downregulated.

**Figure 8:**
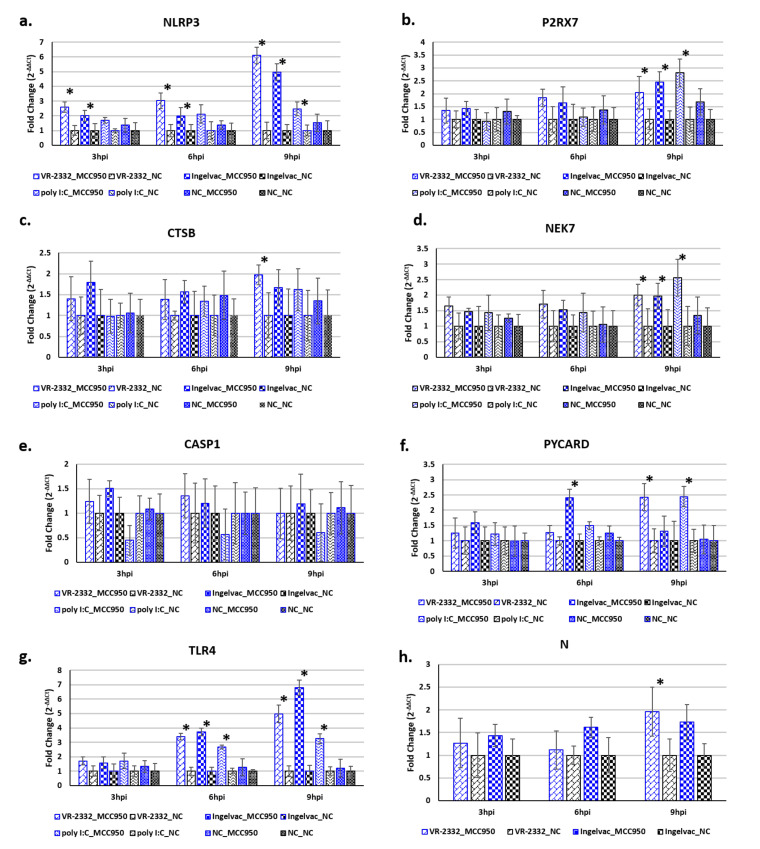
Inhibition of NLRP3 inflammasome activity in alveolar macrophages is beneficial to PRRSV replication. PAMs (*n* = 3) were treated with the NLRP3 inflammasome inhibitor MCC950 at a final concentration of 10 μM or mock-treated. One-hour post-treatment PAMs were either infected with VR-2332 (M.O.I. 1), Ingelvac MLV (M.O.I. 1), transfected with 1 μg of poly I:C (known NLRP3 inflammasome activator) or mock infected. Expression levels of NLRP3 inflammasome-associated cellular genes (**a**–**g**) and PRRSV RNA levels (**h**) were assayed at 3, 6, and 9 hpi using RT-qPCR. Values are provided as fold differences in gene expression levels between time-matched, MCC950-treated PAMs and mock-treated PAMs with same viral/poly I:C treatment, error bars denote SD for each group. * significantly (*p* < 0.05) different expression levels between time-matched, MCC950-treated PAMs and mock-treated PAMs with same viral/poly I:C treatment. VR-2332_MCC950: PAMs treated with 10 μM MCC950 for 1h then infected with VR-2332 (M.O.I. 1). VR-2332_NC: Mock-treated PAMs infected with VR-2332 (M.O.I. 1). Ingelvac MLV_MCC950: PAMs treated with 10 μM MCC950 for 1 h then infected with Ingelvac MLV (M.O.I. 1). Ingelvac MLV_NC: Mock-treated PAMs infected with Ingelvac MLV (M.O.I. 1). poly I:C_MCC950: PAMs treated with 10 μM MCC950 for 1 h then transfected with poly I:C (1 μg). poly I:C_NC: Mock-treated PAMs transfected with poly I:C (1 μg). NC_NC: Mock-treated, mock-infected PAMs.

RNA-seq analysis suggested that many NLRP3 inflammasome-associated genes were differentially expressed in PRRSV-infected PAMs (Figure 7). Treatment of PAMs with a NLRP3 inflammasome-specific inhibitor (MCC950), resulted in significantly (*p* < 0.05) higher mRNA levels of NLRP3 at 3, 6, and 9 hpi in both VR-2332-infected and Ingelvac MLV-infected cells (Figure 8a). *P2RX7* expression was significantly (*p* < 0.05) higher in MCC950-treated PAMs infected with either VR-2332 or Ingelvac-MLV at 9 hpi (Figure 8b). *CTSB* mRNA levels were significantly (*p* < 0.05) higher in VR-2332-infected PAMs treated with MCC950 vs. mock-treated cells (Figure 8c). *NEK7* expression was significantly (*p* < 0.05) higher in MCC950-treated PAMs infected with either VR-2332 or Ingelvac MLV at 9 hpi (Figure 8d). The mRNA levels of *CASP1* did not significantly differ between any of the treatment groups (Figure 8e). *PYCARD* expression was significantly (*p* < 0.05) higher in MCC950-treated PAMs infected with Ingelvac MLV at 6 hpi vs. mock-treated Ingelvac MLV-infected PAMs and VR-2332 at 9 hpi vs. mock-treated VR-2332-infected PAMs (Figure 8f). *TLR4* expression levels were significantly (*p* < 0.05) higher in both VR-2332-infected and Ingelvac MLV-infected MCC950-treated PAMs at 6 and 9 hpi vs. mock-treated infected PAMs (Figure 8g). Viral RNA levels of VR-2332 were significantly (*p* < 0.05) higher in MCC950-treated PAMs at 9 hpi than mock-treated PAMs (Figure 8h). Ingelvac MLV RNA levels also tended (*p* > 0.05) to be higher in MCC950-treated PAMs at 9 hpi vs. mock-treated PAMs (Figure 8h). A significant reduction in mature IL1B secretion into culture supernatants was confirmed by ELISA (Figure S2).

**Figure 9:**
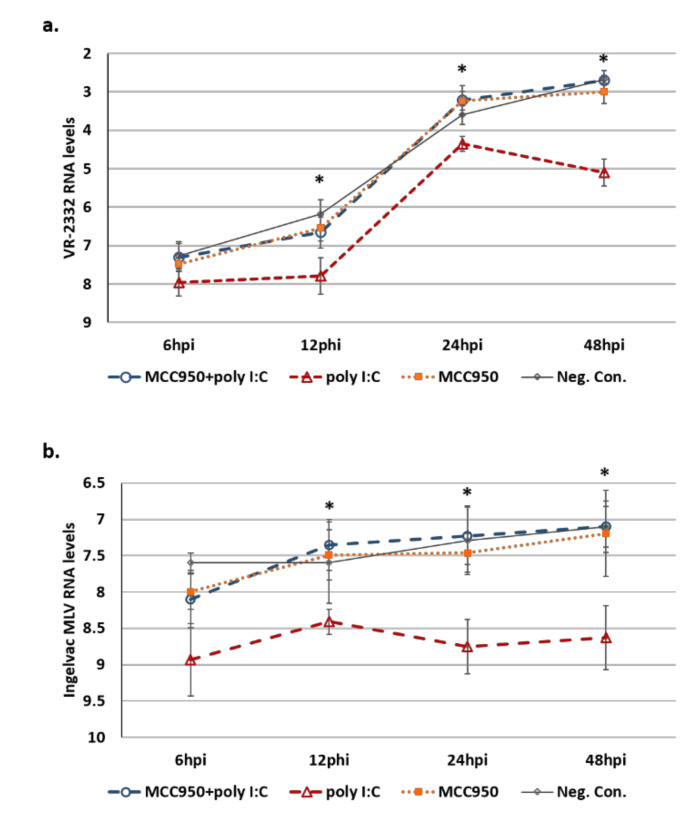
Treatment of PAMs with cell culture supernatants from NLRP3 inflammasome activated PAMs reduces PRRSV replication. PAMs (*n* = 3) were treated with the NLRP3 inflammasome inhibitor MCC950 at a final concentration of 10 μM or mock-treated. One hour post-treatment PAMs were transfected with 1 μg of poly I:C (known NLRP3 inflammasome activator) or mock transfected. Twenty-four hours post-transfection cell culture supernatants were collected and used to treat a second group of pig-matched PAMs. One hour-post treatment PAMs were either infected with (**a**) VR-2332 (M.O.I. 1), or (**b**) Ingelvac MLV (M.O.I. 1). PRRSV RNA levels were assayed at 6, 12, 24 and 48 hpi using RT-qPCR. Values are provided as the mean normalized viral RNA levels (N expression values-RPL4 expression values), error bars denote SD for each group. * significantly (*p* < 0.05) different viral RNA levels between time-matched, PAMs treated with MCC950 treated and poly I:C transfected cell culture supernatants (blue line) and PAMs treated with poly I:C transfected cell culture supernatants (red line). MCC950+poly I:C: PAMs treated with cell culture supernatants from PAMs subjected to MCC950 treatment and poly I:C transfection. poly I:C: PAMs treated with cell culture supernatants from PAMs subjected to poly I:C transfection only. MCC950: PAMs treated with cell culture supernatants from PAMs subjected to MCC950 treatment only. Neg. Con.: PAMs treated with cell culture supernatants from PAMs subjected to mock treatment/transfection.

In a second experiment PAMs were MCC950 treated or mock treated and then transfected with poly I:C to induce the NLRP3 inflammasome response. Significant reduction in mature IL1B secretion was confirmed by ELISA (Figure S2). NLRP3 inflammasome-dependent cytokine secretion in response to poly I:C transfection will be impaired in MCC950-treated PAMs but not in mock-treated cells. A second group of PAMs were then treated with pig-matched cell culture supernatants from these NLRP3 inflammasome inhibited or NLRP3 inflammasome activated PAMs prior to infection with either VR-2332 or Ingelvac MLV. Though not statistically significant (*p* > 0.05), at 6 hpi, PAMs treated with cell culture supernatants from poly I:C transfected PAMs had lower viral RNA levels (1.57-fold lower VR-2332 RNA levels; 1.78-fold Ingelvac MLV RNA levels) compared to PAMs treated with cell culture supernatants from MCC950-treated/poly I:C-transfected PAMs (Figure 9). At 12 hpi both VR-2332 RNA levels (2.18-fold; *p* < 0.05) and Ingelvac MLV RNA levels (2.47; *p* < 0.05) were significantly lower in PAMs pretreated with cell culture supernatants from poly I:C transfected PAMs compared to PAMs pretreated with cell culture supernatants from MCC950-treated/poly I:C-transfected PAMs. At 24 hpi VR-2332 RNA levels were 2.20-fold lower (*p* < 0.05) and Ingelvac MLV RNA levels were 2.88-fold lower (*p* < 0.05) in PAMs pretreated with cell culture supernatants from poly I:C transfected PAMs compared to PAMs pretreated with cell culture supernatants from MCC950-treated/poly I:C-transfected PAMs. At 48 hpi VR-2332 RNA levels were 5.22-fold lower (*p* < 0.05) and Ingelvac MLV RNA levels were 2.89-fold lower (*p* < 0.05). Pretreatment of PAMs with cell culture supernatants from PAMs subjected to MCC950 treatment alone or mock treated/transfected did not have any significant impact on viral RNA levels at any of the time points assayed (Figure 9). Typically, IL1B expression by immune cells is low and its production must be triggered by some form of immunological stimulant [18]. This is the likely explanation for the lack of effect seen when PAMs were pretreated with cell culture supernatants from PAMs subjected to MCC950 treatment alone. MCC950, itself, is not known to trigger an immune response, so these PAMs would not be expected to undergo NLRP3 inflammasome activation and subsequent IL1B production. Therefore the IL1B levels in these supernatants would be expected to be similar to those of mock-treated cells. We found that secreted IL1B levels were not significantly different between these groups (Figure S2). Additionally, any residual MCC950 in these cell culture supernatants would be diluted below its effective dosage when added to the secondary PAM cultures and so would have minimal impact on the inflammasome activity of these newly treated cells.

## 4. Discussion

Since its emergence 30 years ago PRRSV has been a major problem for the worldwide swine industry. There are several issues which currently limit the effectiveness of PRRS control strategies. Available vaccines do not provide broad protection or elicit robust PRRSV-specific immunity. It has also been established that PRRSV deploys several antagonistic tactics to evade and actively suppress the host’s immune responses to infection [3]. In order to improve future vaccine designs and disease prevention programs, a better understanding of the immunosignature of PRRSV-infected macrophages is needed. To this end, in the present study, we utilized RNA-seq to develop global profiles of transcriptome changes in PRRSV-infected PAMs to explore the PRRSV immunosignature. As expected, we found that a number of immune signaling networks are altered in PRRSV-infected PAMs, interestingly, among these is the innate immune regulatory system, the NLRP3 inflammasome. The NLRP3 inflammasome is a well-known of branch innate immunity, predominantly expressed in macrophages, triggered by numerous pathogen-associated molecular patterns (PAMPs) and danger-associated molecular patterns DAMPs, which leads to maturation of several key cytokines, including IL1β and IL18, and a specialized type of cell death pyroptosis (Reviewed by [5]). In light of the importance of the NLRP3 inflammasome in innate immunity and its involvement in the antiviral responses [19], we chose to further explore its potential impacts on PRRSV. We utilized an NLRP3 inflammasome inhibitor, MCC950, which specifically and potently blocks NLRP3 inflammasome activation (prevents pro-caspase-1 processing into its mature form) [13] to elucidate the potential impact of inflammasome activity on the PRRSV lifecycle.

Similarly to others, here we found that *IL10* expression is significantly and quickly increased in PAMs upon PRRSV infection (Figure 1, Figure 2 and Figure 3). In macrophages IL-10 production can be triggered via several signaling pathways, including TLR signaling and c-type lectin receptor signaling [20]. In the present study several c-type lectin receptors were significantly (*p* < 0.05) up-regulated by PRRSV infection in PAMs. These lectins include: *CLEC11A* (12 hpi: 2.027 log_2_, 24 hpi: 1.802 log_2_); *CLEC5A* (12 hpi: 1.124 log_2_, 24 hpi: 0.915 log_2_); *CLEC7A* (12 hpi: 1.410 log_2_, 24 hpi: 1.130 log_2_); and *CLEC2B* (12 hpi: 0.715 log_2_). C-type lectin receptors are one branch of pattern recognition receptors (PRRs) which recognize pathogen-associated molecular patterns (PAMPs) to activate innate and adaptive immune responses following pathogenic infections (Reviewed by [21]) and can also trigger a cellular oxidative phosphorylation (OXPHOS) to glycolytic metabolic shift [22]. This increased expression of c-type lectin receptor expression induced in PRRSV-infected macrophages likely contributes to the up-regulation of IL-10 in these cells. Signaling via both CLEC5A and CLEC7A can induce TNF production, which we found to be up-regulated in PRRSV-infected macrophages (Figure 1, Figure 2 and Figure 3). TNF is a well-known multifunctional cytokine secreted by macrophages which plays numerous roles in immunological responses, including regulation of T-cell responses, glucose metabolism, cytokine secretion and apoptosis [23]. CLEC7A signaling also leads to increased expression of other cytokines as well, including IL-1β, IL-23, and IL-10 [24]. Here we found the mRNA levels of *IL-1β* and *IL-23* are significantly higher in PRRSV-infected macrophages compared to mock-infected cells. IL-1β and its related family member IL-1α (also significantly up-regulated in PRRSV-infected PAMs) are both potent pro-inflammatory cytokines, which are produced by activated macrophages in response to pathogenic infections [25]. Initially both of these IL-1 cytokines are produced as inactive proproteins that require processing by caspase 1 to generate active forms of the proteins [26]. This processing requires the activity of the NLRP3 inflammasome [26]. IL-10 is also known to be involved in NLRP3 inflammasome activation by altering metabolic activities in macrophages [27]. Among IL-10’s metabolic activities is the conversion between glycolysis and oxidative phosphorylation (OXPHOS) metabolic processes. Furthermore, knockout of IL-10 in murine macrophages leads to both mitochondrial damage and subsequently dysregulation of NLRP3 inflammasome activity [27]. It was recently shown that macrophages exposed to PAMPs produce elevated levels of several cytokines, including TNF, IL-12p70, IL-6, and IL-10 [24]. PAMP-exposed macrophages also experience a metabolic shift from OXPHOS to glycolysis, resulting in an increase in lactate release, decreased mitochondrial oxygen consumption, and increased mitochondrial reactive oxygen species (ROS) production. IL-10 can function in an autocrine metabolic feedback loop during macrophage activation [28]. It was demonstrated that LPS-treated macrophages undergo increased glycolytic and reduced OXPHOS metabolic processes. This glycolytic flux regulates IL-10 production by mTOR, and in turn IL-10 then regulates nitric oxide (NO) production to limit NO-mediated suppression of OXPHOS. It was suggested that the IL-10 metabolic feedback loop serves to maintain metabolic equilibrium in activated macrophages and disruptions of this loop can alter the balance between proinflammatory and immunosuppressive phenotypes [28].

We found a number of genes associated with NLRP3 inflammasome activity were differentially expressed in PRRSV-infected macrophages (Figure 7). Among the DAMPs which can serve as the activation signal for NLRP3 inflammasome activity is extracellular ATP (Reviewed by [29]). The P2 × 7 receptor (P2RX7) is an ATP-gated ion channel expressed by immune cells, including macrophages [30]. Excessive ATP levels may occur due to increased energy demands and mitochondrial damage in infected and/or apoptotic cells. Binding of ATP activates P2RX7, allowing it to bind to NLRP3 and activate the inflammasome. In a complementary mechanism, detection of ATP-induced changes in potassium (K+) efflux by the NIMA Related Kinase 7 (NEK7) kinase, can also activate NLRP3 inflammasome activity [29]. In the transcriptional profiling of PRRSV-infected PAMs, we found that *P2RX7* was significantly higher in infected cells (12 hpi: 2.162 log_2_). Interestingly, P2RX7 activation has been found to be both beneficial and detrimental to resolving viral infections (Reviewed by [29]). For example, P2RX7 activation decreases Dengue Virus (DENV) viral loads, whereas it increases Hepatitis B Virus (HBV) and HIV replication. This suggests that while P2RX7 activation is an important antiviral innate immune response, viruses have developed mechanisms to subvert it for their own benefit. The NOD-like receptor, NLRX1 can serve as both a positive and negative regulator of NLRP3 inflammasome activity to maintain appropriate levels of NLRP3 inflammasome-induced responses [31]. NLRX1 can recruit the adaptor protein, ASC, to join the inflammasome complex, an essential component for inflammasome activation [31]. However, NLRX1 can also negatively regulate NF-κB signaling [31], the main priming event for NLRP3 inflammasome activation [32]. Here, we found that *NLRX1* mRNA levels were significantly higher in PRRSV-infected PAMs (12 hpi: 1.385 log_2_; 48 hpi: 0.603 log_2_). As with P2RX7, viruses have been found to manipulate NLRX1 activity to their advantage. A siRNA screen found that NLRX1 was required for HIV infection of human macrophages and NLRX1 knock-out (KO) mice had reduced HSV-2 viral loads [33]. This suggests that viruses are able to manipulate the inhibitory functions of NLRX1 to mute antiviral responses, which may explain the increase in NLRX1 expression in PRRSV-infected PAMs.

Owing to the major role the NLRP3 inflammasome plays in innate immunity, we utilized a NLRP3-specific inhibitor to explore its potential involvement in PRRSV infections. As NRLP3 inflammasome activity has been linked to a number of chronic inflammatory diseases, efforts have been undertaken to produce NLRP3 inflammasome inhibitory drugs (Reviewed by [34]). Among these is a diarylsulfonylurea-containing compound termed MCC950 (Reviewed by [13]). It is known that MCC950 works at the post-translational level in a reversible fashion, though the exact mechanism of action of is unknown. However, several studies have suggested that it likely blocks the ATP hydrolysis domain of NLRP3, thus inhibiting cleavage of the pro-caspase-1 protein into its active form [13]. MCC950 is commonly used to study NLRP3 inflammasome functions, as it is both highly specific and potent [34].

In the present study we initially used MCC950 to block NLRP3 inflammasome activity in PAMs to determine its possible role(s) in PRRSV replication and the innate macrophagic responses during the earliest stages of infection, by treating PAMs with MCC950 (10 µM final concentration) prior to PRRSV infection. We found that blocking inflammasome activity did not significantly alter PRRSV RNA levels, at 3 and 6 hpi (Figure 8). However, by 9 hpi PAMs treated with MCC950 had significantly higher (~2-fold, *p* < 0.05) PRRSV VR-2332 RNA levels (measured by N qPCR) than untreated PAMs (Figure 8). The PRRSV Ingelvac MLV vaccine strain also had higher RNA levels in MCC950 treated PAMs at 9 hpi (1.73 fold), but was not statistically significant. The observation that MCC950 inhibition of NLRP3 inflammasome function positively impacts PRRSV RNA levels at 9 hpi suggests that inflammasome-induced proinflammatory responses negatively affect nascent virus production in PAMs, at least in vitro. There could be several plausible explanations for this. It is possible that the increased inflammasome activity in mock-treated cells induced cellular inflammatory stresses and potential caspase-1 dependent apoptotic factors create a less favorable environment for optimal viral machinery function. Conversely, it is also possible that the reduction in IL1β protein maturation in PAMs treated with MCC950 prior to infection reduces the overall immunological response to infection in these cells and creates a more favorable environment for PRRSV replication, than in mock-treated PAMs, which should have fully functional NLRP3 inflammasome activity.

We also found several NLRP3 inflammasome-associated genes were differentially expressed in PRRSV-infected PAMs treated with MCC950 versus PRRSV-infected mock-treated PAMs (Figure 8b). Among these is *P2RX7*, which was significantly higher in MCC950-treated PAMs, infected with either PRRSV VR-2332 or the Ingelvac-MLV vaccine, and in those transfected with poly I:C, but not between PAMs only treated with MCC950 and untreated PAMs (Figure 8). This could be indicative of disruption of the downstream feedback loops after PRR stimulation. It is also possible that, as MCC950 is thought to block the ATP hydrolysis motif of the inflammasome therefore preventing it from utilizing ATP, this could result in a steady excess of ATP in MCC950-treated cells. This could, in turn, maintain heightened PRR signaling in MCC950-treated cells, which could also explain the higher *NLRP3* mRNA levels in MCC950-treated PAMs (Figure 8a). Disruption of NLRP3′s ability to process pro-caspase-1 will prevent all downstream signals, so the cells may not recognize that the inflammasome has been activated and will continue to prime it. We also found that the expression of the lysosomal cysteine protease Cathespin B (*CTSB*) was moderately higher in PRRSV-infected PAMs treated with MCC950 vs. untreated PAMs at 9 hpi (Figure 8c). CTSB is released into the cytosol upon lysosomal destabilization, where it then processes a number of proproteins, including the caspases, CASP3, CASP9 and CASP11, the latter of which likely functions in TLR4-independent, non-canonical NLRP3 inflammasome activation [35]. Additionally, P2RX7 is able to facilitate the release of activated CTSB, independent of its role in inflammasome activation [36]. Under certain conditions, CTSB may process limited amounts of pro-IL1β independent of the NLRP3 inflammasome [37]. As CTSB is able to process pro-IL1β, albeit to a far lesser extent, independently of the NLRP3 inflammasome, it is possible that the increase in *CTSB* expression may be a compensatory response to the lack of inflammasome functionality in MCC950-treated cells, perhaps triggered by increased danger signals, e.g., ATP and/or viral RNA, in these cells. Taken together these results suggest that PRRSV infections, by both pathogenic and vaccines strains, can quickly induce NLRP3 inflammasome activation.

Poly I:C transfection is a potent inducer of NLRP3 inflammasome activation [16]. To further explore the involvement of NLRP3 inflammasome activity in PRRSV infections, in a second experiment, we used poly I:C transfection to first induce NLRP3 inflammasome activation in MCC950-treated or mock-treated PAMs. As discussed above, MCC950 prevents NLRP3 inflammasome processing of pro-caspase-1 into its active form. Therefore, PAMs treated with MCC950 will not secrete the NLRP3-dependent cytokine milieu from the cell, including the mature forms of IL-1β and IL-18, unlike mock-treated cells, in response to poly I:C transfection. We then used cell culture supernatants from these NLRP3 inflammasome-inhibited (MCC950-treated/poly I:C-transfected) or NLRP3 inflammasome-activated (mock-treated/poly I:C-transfected) PAMs to treat pig-matched PAMs prior to PRRSV infection. We found that PRRSV RNA levels, concerning both VR-2332 and Ingelvac-MLV, were significantly (*p* < 0.05) lower in PAMs treated with cell culture supernatants from NLRP3 inflammasome activated PAMs compared to culture supernatants from NLRP3 inflammasome inhibited PAMs (Figure 9). At 24 hpi PAMs pretreated with cell culture supernatants from NLRP3 inflammasome-activated PAMs had ~2-fold lower (*p* < 0.05) VR-2332 viral RNA levels than PAMs pretreated with cell culture supernatants from NLRP3 inflammasome-inhibited PAMs. At 48 hpi VR-2332 viral RNA levels were ~5-fold lower (*p* < 0.05) in PAMs pretreated with cell culture supernatants from NLRP3 inflammasome-activated PAMs. At 24 and 48 hpi PAMs treated with cell culture supernatants from NLRP3 inflammasome-activated PAMs had ~3-fold lower (*p* < 0.05) Ingelvac MLV viral RNA levels. This provides additional support for a role for NLRP3 inflammasome activation in the macrophagic anti-PRRSV response and suggests that PRRSV is likely sensitive to the effects of the NLRP3 inflammasome cascade.

## 5. Conclusions

It has become clear that PRRS severity and host resistance to and clearance of PRRSV is predicated on a complex collection of factors. These include host factors such as genetic background, age, health status, and immune responsiveness and viral factors such as strain virulence and viral host immune suppression strategies. A better understanding of the complex interactions between PRRSV and its target cell the alveolar macrophage is needed to develop more effective PRRS preventatives. In the present study, transcriptome profiling revealed that PRRSV quickly induces marked changes in the immune signature and metabolic profile of infected cells. Among these is the innate immune regulatory pathway induced by NLRP3 inflammasome activation. Chemical inhibition of NLRP3’s ability to proteolytically process the pro-caspase-1 protein into its mature form and thus preventing downstream signaling, led to increased levels of PRRSV RNA by 9 hpi. Furthermore, pretreatment of PAMs with cell culture supernatants from PAMs under NLRP3 inflammasome activation significantly reduced PRRSV RNA levels. This suggests that PRRSV is sensitive to NLRP3 signaling, which could provide a new avenue for future prophylactic development. Further elucidation of the specific involvement of these changes in PRRSV pathogenesis and host immunity will be beneficial to novel vaccine design and disease prevention strategies.

## Figures and Tables

**Table 1:** The top canonical pathways altered in Porcine Reproductive and Respiratory Syndrome Virus (PRRSV)-infected porcine alveolar macrophages (PAMs) in vitro.

Time-Point Post Infection	Canonical Pathway
12 hpi	LXR/RXR Activation
	Phagosome Formation
	LPS/IL-1 mediated Inhibition of RXR Function
	Production of Nitric Oxide and Reactive Oxygen Species in Macrophages
	IL-6 Signaling
	CXCR4 Signaling
	IL-12 Signaling and Production in Macrophages
	NRF2-mediated Oxidative Stress Response
	Role of Pattern Recognition Receptors in Recognition of Bacteria and VirusesInflammasome pathway
	LPS-stimulated MAPK Signaling
	NF-κB Activation by Viruses
	TR/RXR Activation
	IL-10 Signaling
	IL-1 Signaling
	NF-κB Signaling
24 hpi	LXR/RXR Activation
	TNFR2 Signaling
	IL-10 Signaling
	Toll-like Receptor Signaling
	Production of Nitric Oxide and Reactive Oxygen Species in Macrophages
	IL-12 Signaling and Production in Macrophages
	TNFR1 Signaling
	NF-κB Activation by Viruses
	Acute Phase Response Signaling
	Activation of IRF by Cytosolic Pattern Recognition Receptors
	IL-6 Signaling
	Phagosome Formation
	MIF Regulation of Innate Immunity
	iNOS Signaling
	NF-κB Signaling
	IL-1 Signaling
	CCR5 Signaling in Macrophages
	Inflammasome pathway
48 hpi	Role of Pattern Recognition Receptors in Recognition of Bacteria and Viruses
	Role of RIG1-like Receptors in Antiviral Innate Immunity
	TNFR1 Signaling
	Activation of IRF by Cytosolic Pattern Recognition Receptors
	Toll-like Receptor Signaling
	Role of PI3K/AKT Signaling in the Pathogenesis of Influenza
	Communication between Innate and Adaptive Immune Cells
	IL-6 Signaling
	NF-κB Signaling
	Role of JAK family kinases in IL-6-type Cytokine Signaling
	Role of PKR in Interferon Induction and Antiviral Response
	MIF Regulation of Innate Immunity
	iNOS Signaling
	Role of Cytokines in mediating Communication between Immune Cells
	IL-23 Signaling Pathway
	IL-10 Signaling
	IL-1 Signaling
	CCR5 Signaling in Macrophages
	LPS-stimulated MAPK Signaling
	NF-κB Activation by Viruses
	PI3K Signaling in B Lymphocytes
	Production of Nitric Oxide and Reactive Oxygen Species in Macrophages

LXR/RXR: Liver X Receptor/Retinoid X Receptor; LPS/IL-1: Lipopolysaccharide/Interleukin-1; CXCR4: C-X-C chemokine receptor type 4; NRF2: Nuclear factor erythroid 2; MAPK: Mitogen-activated protein kinase; NF-κB: Nuclear factor kappa B; IFN: Interferon regulatory factor; TR/RXR: Thyroid hormone/Retinoic acid receptors; MIF: Macrophage migration inhibitory factor; iNOS: Inducible nitric oxide synthase; CCR5: C-C chemokine receptor type 5; RIG1: Retinoic Acid Inducible Gene 1; TNFR2: Tumor necrosis factor receptor 2; IRF: IFN regulatory factor; PI3K/AKT: Phosphoinositide 3-kinase/Protein kinase B; JAK: Janus kinase; PKR: RNA-dependent protein kinase.

## Supplementary Materials

The following are available online at https://www.mdpi.com/1999-4915/12/11/1299/s1, Figure S1: Expression pattern confirmation for a random selection of differentially expressed genes from RNA-seq via RT-qPCR, Figure S2: Confirmation of MCC950’s ability to prevent IL1B secretion in porcine alveolar macrophages, Table S1: Primers used in this study.
