# Supplementary material for: Transcriptional Immune Signatures of Alveolar Macrophages and the Impact of the NLRP3 Inflammasome on Porcine Reproductive and Respiratory Syndrome Virus (PRRSV) Replication
Source: Viruses. 2020 Nov 12;12(11):1299. doi: 10.3390/v12111299 (PMC7696364; doi:10.3390/v12111299)
Supplement: Supplementary file 1 [file viruses-12-01299-s001.zip › Figure S1.pdf]

Figure S1

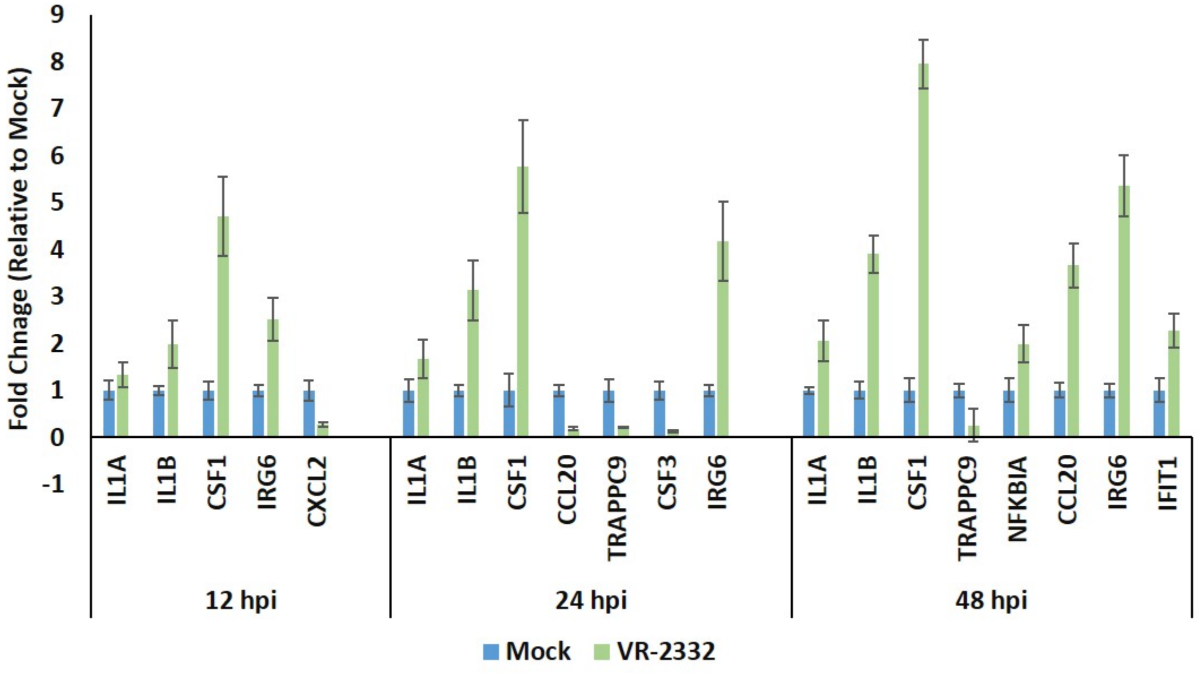

Figure S1. Expression pattern confirmation for a random selection of differentially expressed genes from RNA-seq via RT-qPCR. PAMs (n=3) were either infected with PRRSV strain VR-2332 or mock infected and total RNA was collected at 12h, 24h, or 48h and subjected to RT-qPCR. All values are provided as the fold change in PRRSV-infected PAMs relative to mock-infected PAMs.
