# Supplementary material for: Transcriptional Immune Signatures of Alveolar Macrophages and the Impact of the NLRP3 Inflammasome on Porcine Reproductive and Respiratory Syndrome Virus (PRRSV) Replication
Source: Viruses. 2020 Nov 12;12(11):1299. doi: 10.3390/v12111299 (PMC7696364; doi:10.3390/v12111299)
Supplement: Supplementary file 1 [file viruses-12-01299-s001.zip › Figure S2.pdf]

Figure S2

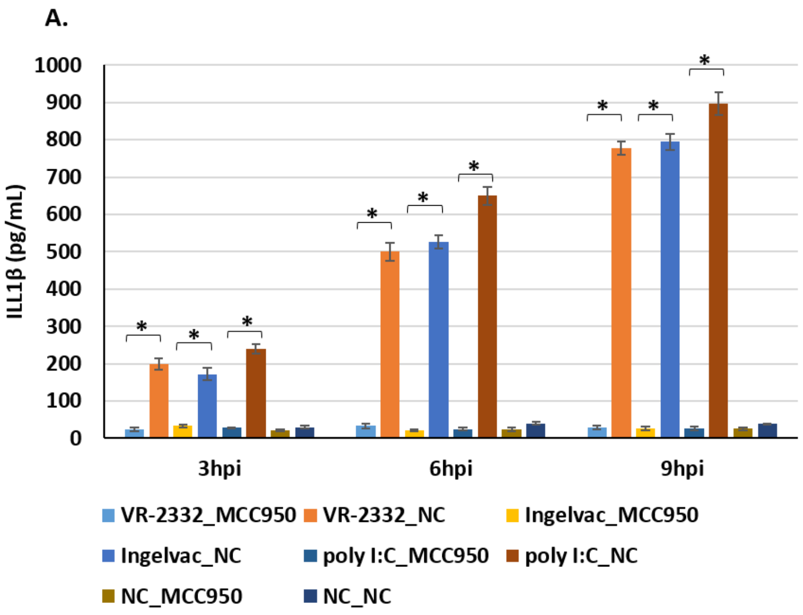

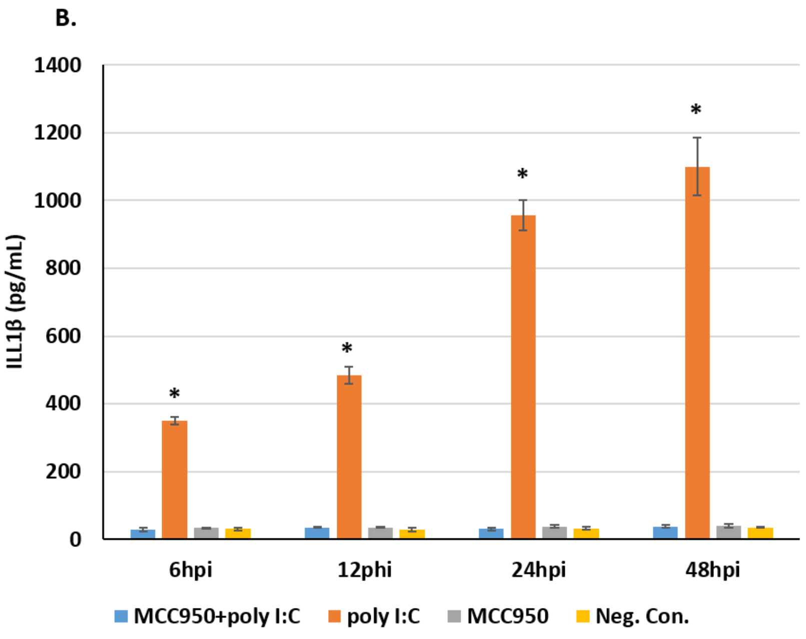

Figure S2. Confirmation of MCC950's ability to prevent IL1B secretion in porcine alveolar macrophages. To confirm the ability of MCC950 to prevent the release of IL1B from PAMs *in vitro*, the levels of IL1B secreted into the cell culture supernatants were measured using an enzyme-linked immunosorbent assay kit (ELISA) for porcine IL1B (MyBioSource, San Diego,

CA, USA), following the manufacturer's instructions. Each sample was assayed in duplicate. Values are provided as average levels (pg/mL) of mature IL1B present in culture supernatants, error bars denote SD for each group. Panel (A.) represented secreted IL1B levels in the PAM cultures from the first inflammasome inhibition experiment (Figure 8 in the text) and Panel (B.) respresents IL1B levels in the PAM cultures from the second inflammasome inhibition study (Figure 9 in the text). (A.) PAMs (n=3) were treated with the NLRP3 inflammasome inhibitor MCC950 at a final concentration of 10μM or mock-treated. One hour post-treatment PAMs were either infected with VR-2332 (M.O.I 1), Ingelvac MLV (M.O.I.1), transfected with 1μg of poly I:C (known NLRP3 inflammasome activator) or mock infected. *significantly ($p<0.05$) different expression IL1B levels between time-matched, MCC950-treated PAMs and mock-treated PAMs with same viral/poly I:C treatment. Panel legend: VR-2332_MCC950: PAMs treated with 10μM MCC950 for 1hr then infected with VR-2332 (M.O.I. 1). VR-2332_NC: Mock-treated PAMs infected with VR-2332 (M.O.I. 1). Ingelvac MLV_MCC950: PAMs treated with 10μM MCC950 for 1hr then infected with Ingelvac MLV (M.O.I. 1). Ingelvac MLV_NC: Mock-treated PAMs infected with Ingelvac MLV (M.O.I. 1). poly I:C_MCC950: PAMs treated with 10μM MCC950 for 1hr then transfected with poly I:C (1μg). poly I:C_NC: Mock-treated PAMs transfected with poly I:C (1μg). NC_NC: Mock-treated, mock-infected PAMs. (B.) PAMs (n=3) were treated with the NLRP3 inflammasome inhibitor MCC950 at a final concentration of 10μM or mock-treated. One hour post-treatment PAMs were transfected with 1μg of poly I:C (known NLRP3 inflammasome activator) or mock transfected. Twenty-four hours post-transfection cell culture supernatants were collected and used to treat a second group of pig-matched PAMs. Panel legend: MCC950+poly I:C: PAMs treated with cell culture supernatants from PAMs subjected to MCC950 treatment and poly I:C transfection. poly I:C: PAMs treated with cell culture supernatants from PAMs subjected poly I:C transfection only. MCC950: PAMs treated with cell culture supernatants from PAMs subjected to MCC950 treatment only. Neg. Con.: PAMs treated with cell culture supernatants from PAMs subjected to mock treatment/transfection. *PAMs transfected with poly I:C alone had significantly higher ($p<0.05$) IL1B levels than the other groups.
